# Benign Lymphoid Hyperplasia of the Tongue Base Causing Upper Airway Obstruction

**DOI:** 10.1155/2011/625185

**Published:** 2011-09-29

**Authors:** Noah B. Sands, Marc Tewfik

**Affiliations:** Department of Otolaryngology-Head and Neck Surgery, Royal Victoria Hospital, McGill University, 687 Pine Avenue West E3-37, Montreal, QC, Canada H3A 1A1

## Abstract

Severe benign lymphoid hyperplasia (LH) is unusual in the head and neck region, but the diagnosis of LH is of clinical importance as it may be confused with malignant lymphoma, both on clinical examination and pathologically. While the etiology is poorly understood, a number of previous theories exist, which are included here in the context of a literature review. In this paper we present a case of severe pharyngeal lymphoid hyperplasia causing airway obstruction and requiring tracheotomy and subsequent surgical debulking.

## 1. Case History

A 64-year-old African Canadian female with a history of urinary incontinence was admitted for an elective bladder suspension procedure by the gynaecology service in our institution. At the time of induction, our service was called emergently due to failed intubation related to a “pharyngeal mass.” 

Upon examination with direct laryngoscopy a large, multiloculated, exophytic mass was identified, emanating from the oropharynx and extending distally to the level of the supraglottis, occupying >90% of the upper aerodigestive tract. The obstructive lesion was biopsied, and specimens were sent fresh for lymphoma histopathology protocol. The airway was subsequently secured, and the procedure was undertaken. Review of the preoperative anaesthesia records revealed no features of airway obstruction nor B symptoms on clinical history. 

Postoperatively the patient was deemed unsafe for extubation and transferred to the intensive care unit while placed on high-dose intravenous dexamethasone. 

**Figure 1:**
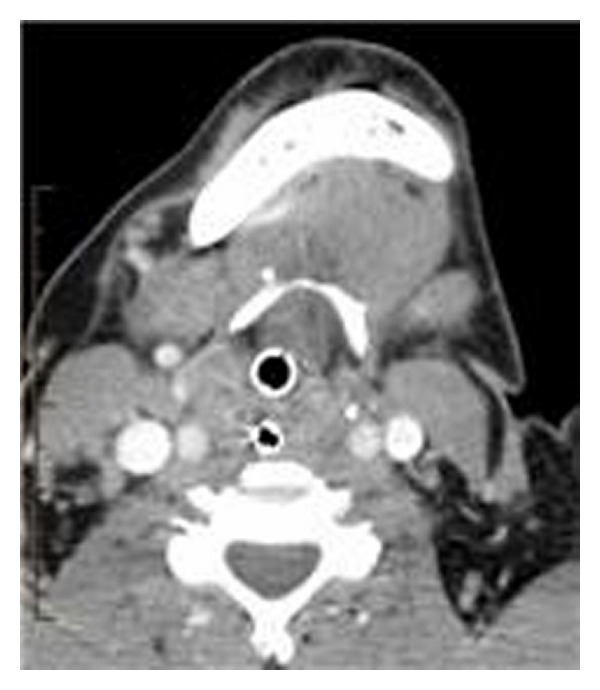
CT scan in the axial plane revealing near-complete airway obstruction at the level of the oropharynx.

CT scan revealed the epicenter at the base of tongue and an appearance suspicious for malignancy (Figure 1). There was no cervical adenopathy, and CT of the thorax and abdomen was negative. HIV serology was negative. Final pathology was determined on postoperative day (POD) 2 to be benign follicular/intrafollicular lymphoid hyperplasia characterized by polyclonal lymphoid proliferation with an inflammatory background. Immunohistochemistry was negative for lymphoma. 

Open tracheotomy was performed on POD 3 due to the absence of a leak, and biopsies were again performed, which ultimately revealed the equivalent benign pathologic findings. 

The patient was kept on a three-week course of tapering prednisone and proton-pump inhibitors. Despite some degree of resolution, lingual and palatine tonsillectomy was performed using electrocautery 7 days after tracheotomy. This procedure was carried out under general anesthetic in the form of a modified adenotonsillectomy, using a Boyle Davis gag for exposure and a combination of monopolar cautery for the palatine tonsils and suction cautery for subtotal ablation of the lingual tonsils. The patient was decannulated and discharged home 14 days after tracheotomy. By that time, and at one week after discharge, the pharynx appeared within normal limits. 

## 2. Discussion

Follicular lymphoid hyperplasia (FLH) is an uncommon benign entity related to a rapid increase in the abundance of lymphocytes contained within or outside of lymph nodes. It has been historically referred to as reactive lymphoid hyperplasia or pseudolymphoma [1]. This entity was first described in 1973 by Adkins. and has since been primarily reported in the skin, breasts, gastrointestinal tract, lungs, and nasopharynx [2]. The majority of existing head and neck reports are of hyperplasia in the oral cavity, namely, of the mucosa overlying the hard palate, and are limited to the dental and pathology literature [3]. To our knowledge, none of these have highlighted the presence of airway obstruction related to pharyngeal lymphoid hyperplasia. 

The diagnosis of FLH is of clinical importance as it may be confused with malignant lymphoma, both on clinical examination and histopathology. The etiology is poorly understood, although some authors have postulated a relationship with chronic irritation (i.e., reflux, poorly fitting dentures, etc.) or a reactive lymphoid proliferation to an unknown antigenic stimulation [2]. While an association with bacterial infection has not been clearly identified, one aggressive case of FLH has been linked to the presence of Epstein-Barr virus, causing clonal *arrangement* (expansion) in the local tissue DNA [4]. 

LH most commonly affects older patients, with a mean age of 61 and female-to-male ratio of nearly 3 : 1. It tends to present as a unilateral, painless, slow-growing, nonulcerated mass. The mean size is 2.5 cm in the literature (range 1–5 cm). Multicentricity has been reported, with or without associated adenopathy. Clinical and laboratory investigations are routinely negative [2]. 

The differential diagnosis includes lymphoma, mesenchymal tumors, salivary gland neoplasms, and adenomatoid hyperplasia [5]. Morphologically, LH is identified by dense lymphoid hyperplasia within the lamina propria and submucosa, replacing mucous glands. Polyclonal lymphoid proliferation with immunohistochemistry stains for kappa or lambda light chains are diagnostic. Pathology may also show indistinct germinal centres leading to erroneous diagnosis of follicular lymphoma [3]. 

Surgical debulking/excision is the treatment of choice. External beam radiation has been successful in a single case [6]. A minority of patients develop local recurrence. Spontaneous regression has also been reported. Only one widely disseminated case has been referenced, which involved cervical nodes, major salivary glands, orbits, and mediastinum [4]. No progression to malignancy has been reported, although one multisite case within the oral cavity was found to represent MALT-type lymphoma [1]. 

Severe benign LH is unusual in the head and neck region, but the diagnosis should be entertained on the part of the clinician both clinically and histologically when lymphoma is suspected—particularly in the oral cavity.
